# Impact of motion management strategies on abdominal organ at risk delineation for magnetic resonance-guided radiotherapy

**DOI:** 10.1016/j.phro.2024.100650

**Published:** 2024-09-16

**Authors:** Mairead Daly, Lisa McDaid, Carmel Anandadas, Andrew Brocklehurst, Ananya Choudhury, Alan McWilliam, Ganesh Radhakrishna, Cynthia L. Eccles

**Affiliations:** a Division of Cancer Sciences, Faculty of Medicine Biology & Health, The University of Manchester, Manchester M13 9PL, United Kingdom; b Department of Radiotherapy, The Christie Hospitals NHS Foundation Trust, Manchester M20 4BX, United Kingdom; c Department of Clinical Oncology, The Christie Hospitals NHS Foundation Trust, Manchester M20 4BX, United Kingdom; d Department of Medical Physics and Engineering, The Christie Hospitals NHS Foundation Trust, Manchester M20 4BX, United Kingdom

**Keywords:** Motion management, MR-guided radiotherapy, SABR, SBRT, IGRT

## Abstract

**Background and purpose:**

The impact of respiratory motion management strategies for abdominal radiotherapy, such as abdominal compression (AC) and breath hold (BH), on abdominal organ at risk (OAR) delineation on magnetic resonance imaging (MRI) is unknown. This feasibility study compared the inter- and intra- observer delineation variation on MRI acquired with AC, BH for three critical abdominal OAR.

**Materials and methods:**

T2-weighted (W) 3D MRI in free-breathing (FB) and with AC, and T1W 3D mDixon exhale BH were acquired. Four observers blinded to motion management strategy used, delineated stomach, liver, and duodenum on all MRI. One case per strategy was repeated over 6 weeks later to quantify intra-observer variation. Simultaneous truth and performance level estimation (STAPLE) contours for each OAR were generated, median and IQR mean distance to agreement (mDTA) and maximum Hausdorff distance (HD) between observer and STAPLE contours were calculated. Observers scored organ visibility on each MRI using a four-point Likert scale.

**Results:**

A total of 27 scans including repeats were delineated. Pooled mDTA for all OARs was 1.3 mm (0.5 mm) with AC, 1.4 mm (1.0 mm) with BH, and 1.3 mm (0.5 mm) in FB. Intra-observer mDTA was highest for all organs in FB with 10.8 mm for duodenum, 1.8 mm for liver, and 2.7 mm for stomach. The pooled mean perceptual quality score value was highest for AC across organs.

**Conclusions:**

No motion management strategy demonstrated superior similarity across OAR, emphasizing the need for personalised approaches based on individual clinical and patient factors.

## Highlights

• The impact of motion management strategies on delineation variation was assessed.
• No differences were seen in inter-observer variation between strategies.
• Overall, perceptual image quality scores were highest with abdominal compression.

## 1 Introduction

Radiotherapy, the use of high-energy ionising radiation [1] remains one of the most effective treatments for cancer [2]. Despite its longstanding use, radiotherapy for abdominal cancers has faced controversy due to potential toxicity [3], and unchanged survival benefit [4]. The challenge of treating abdominal cancers is compounded by the presence of mobile, dose sensitive structures, such as the duodenum and stomach, which complicates abdominal stereotactic ablative body radiotherapy (SABR). Strategies to address respiratory motion include abdominal compression (AC) or breath hold (BH). AC devices apply gentle pressure against the abdomen and help reduce motion caused by breathing, while BH techniques involve the patient holding their breath briefly multiple times throughout treatment delivery [5]. Both methods aim to enhance the precision of radiation delivery, and potentially reduce treatment toxicity due to reduced treatment volumes [6], [7]. Alternatively, when AC or BH are not feasible due to patient or technological factors, treatment in free-breathing (FB) with an internal target volume (ITV) is used [8]. The ITV approach with either AC or FB are most commonly used due to relative ease of implementation, with BH often presenting technological challenges [9]. Magnetic resonance imaging (MRI) is attractive for adaptive radiotherapy due to superior soft-tissue definition of abdominal organs at risk (OARs) than on computed tomography (CT) [10]. However, the impact of various motion management strategies on MRI quality, and consequently, on the accuracy of abdominal OAR delineation, remains unclear.

Motion artefacts, such as blurring or ghosting, can impair MRI quality [11], potentially leading to inaccuracies in delineation, requiring larger planning margins [12]. There are various strategies to evaluate image quality, including geometric and qualitative methods [13]. Quantitative methods such as root mean square error (RMSE) and structural similarity index (SSIM) are often used. However, these require repeat imaging for ground truth comparison, and do not always predict subjective, or perceptual, image quality or utility in the diagnostic setting [14].

Delineation variation for radiotherapy planning, particularly for the target, can introduce systematic uncertainties into the treatment workflow that can lead to undesirable dosimetric effects throughout a course of treatment [12]. Inter- and intra-observer delineation variation in the abdomen has been evaluated previously for upper abdominal targets using CT [15], [16], [17], [18]. The addition of MRI has been shown to reduce inter-observer variation in pancreatic cancer target delineation, likely due to improved soft tissue visibility of tumours [19]. However, there are few data on the variation for abdominal OAR, and no published studies assessing the impact motion management strategies have on delineation variation.

The aim of this study was to assess the feasibility of evaluating inter-observer variation of OAR delineation across motion management strategies, and to quantify inter-observer variability in delineation of three critical abdominal OAR (liver, duodenum, stomach) with AC, BH and in FB. Finally, the aim was to evaluate the relationship between perceptual quality of MRI and inter-observer variability in OAR delineation.

## 2 Materials and methods

### 2.1 Imaging details

Six participants, five of whom were undergoing radiotherapy to abdominal tumours, were recruited with informed consent to an institutional ethics-board-approved imaging study (20 January 2021, 20/WA/0353) [20]. All patients were imaged on 1–2 occasions with three motion management scenarios at each session: FB, AC and audio-coached voluntary end exhale BH. MRIs were acquired on a 1.5 T Unity magnetic resonance linear accelerator (MR Linac, Elekta AB, Stockholm, Sweden). To assess the influence of motion on image quality, images were not reconstructed using motion correction. 3D T2 weighted (W) turbo spin-echo (TSE) images were acquired for FB and AC as they are available within the clinical Unity MR Linac workflow. T1W 3D mDixon MRI were acquired for BH due to the shorter acquisition time than T2 MRI, allowing acquisition of the abdomen within a single BH and were reconstructed with fat and water in-phase due to similarity in appearance to T2W. A series of ten consecutive BHs of 17.9 s were acquired, with a fixed 30 s gap between acquisitions to allow for patient recovery. The first of each BH series was selected for this study unless significant artefact was present suggesting the participant had not correctly achieved BH the first time. A summary of MRI acquisition parameters is shown in Table 1.

**Table 1:** MR image acquisition parameters for sequences included in this study: free-breathing (FB), abdominal compression (AC) and breath hold (BH). T2W = T2-weighted, TSE = turbo spin-echo.

	**FB, AC**	**BH**
**Acquisition type**	3D	3D
**Weighting**	T2W TSE	T1W mDixon
**Echo time (TE)**	70 ms	TE1: 1.82 ms
		TE2: 4.8 ms
**Repetition time (TR)**	1187 ms	6.9 ms
**Flip angle**	90°	10°
**Field of view (FOV)**	450 × 399 mm	420 × 385 mm
**In-plane resolution**	2.0 × 2.0 mm	3.0 × 3.0 mm
**Slice thickness**	3 mm	3 mm

### 2.2 Scan, OAR, and observer information

**Fig. 1:**
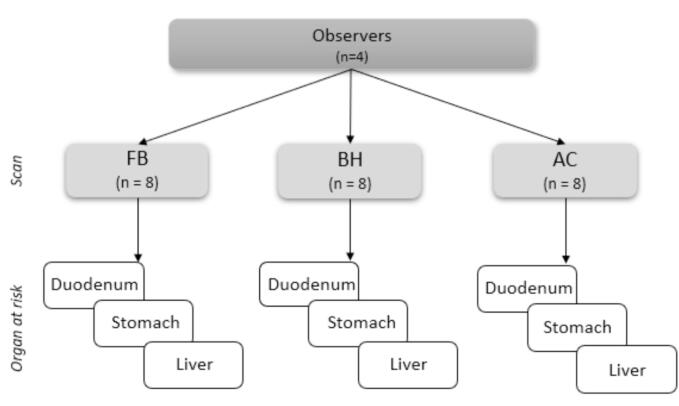
Study schema detailing the number of OAR delineated (excluding repeats), and images per motion management strategy. FB = free-breathing, BH = breath hold, AC = abdominal compression.

**Fig. 2:**
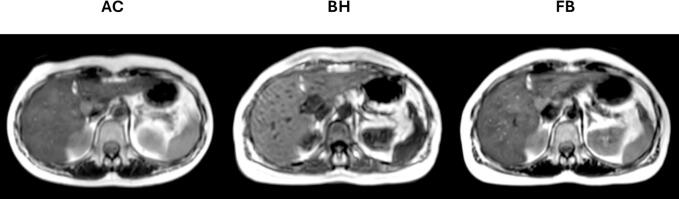
Example of image sequences for abdominal compression (ac, t2w turbo spin-echo), breath hold (bh, in-phase reconstruction mdixon), and free-breathing (fb, t2w turbo spin-echo).

Participants scanned for this study included one non-patient volunteer and five patients receiving abdominal radiotherapy. Median age of participants was 59 years (range, 36–78 years), and a male: female ratio of 2:1. Treatment sites for five patients were liver (n = 4) and pancreas (n = 1). Four observers (two radiation oncologists, two therapeutic radiographers) with clinical expertise in abdominal OAR delineation on MRI were identified within our institution. The median radiotherapy experience of observers was 12 years (range 4–24 years). Scans from a total of eight imaging visits for six participants were included, with two participants attending twice, yielding 24 cases that were delineated by four observers (Fig. 1). To ensure feasibility of the study, three OAR were selected for delineation based on segmentation accuracy results from Noel *et al* [21]: duodenum, stomach, liver (from challenging to less challenging to delineate). One participant had undergone a pancreaticoduodenectomy prior to radiotherapy, resulting in omission of the duodenum contour for their cases. Delineation took place over approximately five months. An example of the three MRI sequences used is shown in Fig. 2.

### 2.3 Delineation

Scans were anonymised and were imported to the treatment planning system (TPS) in a random sequence generated using an online random sequence generator [22] to blind observers to the motion management strategy used. Delineations were performed using the Monaco TPS (V5.51.11, Elekta AB, Stockholm, Sweden). Observers were provided with an MRI consensus atlas paper [23] prior to commencement of the study and instructed that all delineation tools and methods normally used clinically were allowed. Intra-observer variation was assessed by re-delineation of one MRI per motion management strategy a minimum of 6 weeks following the completion of all other contours. Anonymised repeat cases were selected using an online random integer generator [24], and observers were similarly blinded to the motion management strategy used. Volume of delineated contours was recorded. Observers scored their ability to distinguish delineated organs on each MRI using a four-point Likert scale (unable to see, fair, good, excellent) in an Excel (V2407, Microsoft Corporation, Redmond, USA) spreadsheet pre-populated with dropdown lists.

### 2.4 Analysis

In the absence of ground truth contours, baseline simultaneous truth and performance level estimation (STAPLE) contours were generated for each OAR per MRI for geometric analysis using ADMIRE (Elekta AB, Stockholm, Sweden). These, along with observer contours, were imported to RayStation (V11B, RaySearch Laboratories, Sweden) for contour similarity analysis. Descriptive statistics including median and IQR of spatial boundary similarity metrics mean distance to agreement (mDTA) and maximum Hausdorff distance (HD) were calculated. Contour volume for all observers across motion management strategies, and perceptual image quality scores were analysed using descriptive statistics, including median and interquartile range (IQR) in Excel. Spearman's rank correlation tests between perceptual image quality score (PIQS) and mDTA were performed using Prism (V10.1.0, GraphPad Software Inc, Boston, USA), with a p-value of less than 0.05 considered statistically significant for a 5 % probability of Type 1 error. These results were also plotted using Excel. Given the exploratory nature of this study, neither a formal power calculation for sample size determination, nor other statistical testing were performed. Sample size was determined based on available imaging sessions acquired with all three motion management strategies, and observer time constraints.

## 3 Results

### 3.1 Inter and intra-observer delineation variation

For all three organs, pooled median interobserver variation was similar across strategies: with mDTA of 1.3 mm (0.5 mm), 1.4 mm (1.0 mm), and 1.3 mm (0.5 mm) for AC, BH and FB, respectively. The duodenum consistently demonstrated the largest HD across motion management strategies. Results for individual organs are summarised in Table 2. There was no notable difference between volumes for OAR across all three motion management strategies. Intra-observer variation, was highest for all organs in FB, with pooled mDTA≤10.8 mm, compared with ≤3.2 in BH and ≤1.8 in AC.

**Table 2:** Median and interquartile range (IQR) for contour similarity metrics including mDTA (mean distance to agreement) and Hausdorff distance (HD) for all across three motion management strategies: abdominal compression (AC), breath hold (BH) and free-breathing (FB).

		**AC**	**BH**	**FB**
	***Metric (unit)***	***Median (IQR)***		
**Duodenum**	**mDTA (mm)**	1.5 (0.6)	2.2 (1.0)	1.5 (1.3)
	**HD (mm)**	37.3	63.9	2.3
	**Volume (cm^3^)**	46.3 (28.9)	52.1 (17.1)	40.4 (26.7)
**Liver**	**mDTA (mm)**	1.3 (0.5)	1.4 (0.5)	1.3 (0.4)
	**HD (mm)**	2.4	2.4	2.3
	**Volume (cm^3^)**	1374.7 (318)	1377.6 (281.0)	1447.1 (281.7)
**Stomach**	**mDTA (mm)**	1.1 (0.3)	1.3 (0.5)	1.2 (0.6)
	**HD (mm)**	2.7	2.4	2.5
	**Volume (cm^3^)**	152.3 (106.3)	203.2 (44.1)	166.8 (115.9)

### 3.2 Perceptual image quality

**Fig. 3:**
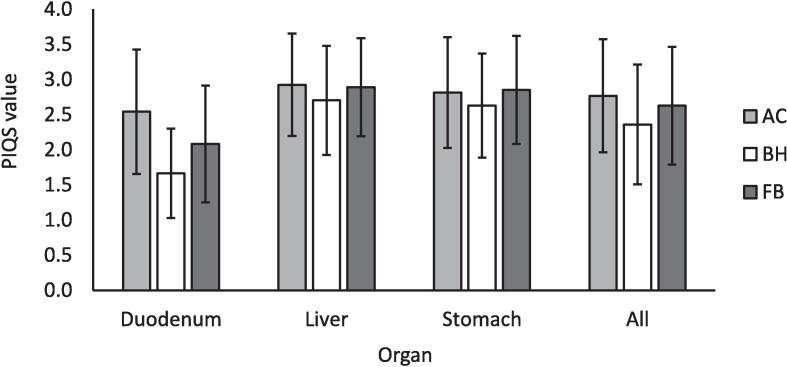
Mean observer perceptual image quality scores (PIQS) for all organ and motion management strategies, as well as pooled for all organs, for three available observers. Error bars show standard deviation. FB=free-breathing, BH=breath hold, AC=abdominal compression.

**Fig. 4:**
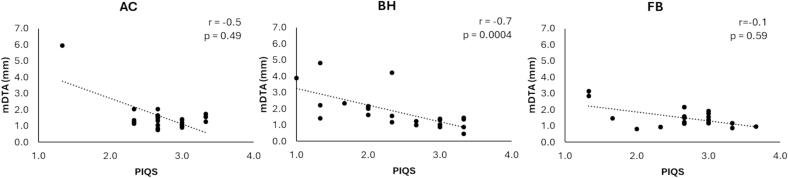
Plots showing correlation between perceptual image quality score (PIQS) and contour mean distance to agreement (mDTA) for abdominal compression (AC), breath hokld (BH), and free-breathing (FB).

PIQS results from three observers were available, the fourth was lost due to computer hardware failure. For all organs, the pooled mean score value was highest for AC. Breath hold (BH) image quality was scored lowest by observers for each organ and motion management strategy (Fig. 3). The duodenum consistently scored lowest overall across all three organs, and BH lowest across all three motion management strategies. The mode, or most frequently occurring score value was 3 for all organs across all motion management strategies, except for the duodenum on BH, with a mode score of 2. There was a moderate statistically significant relationship between higher image quality score and reduced inter-observer contour variation on BH, and an overall similar trend for image quality score and inter-observer contour variation, although not significant for any other motion management strategy. Spearman's rank correlation coefficient for all organs was −0.15 (*p* = 0.49), −0.67 (*p* < 0.0004) and −0.12 (*p* = 0.59) for the relationship between PIQS and mDTA for AC, BH and FB, respectively (Fig. 4), and −0.18 (*p* = 0.41), −0.68 (*p* = 0.0004) and −0.12 (*p* = 0.59) for the relationship between PIQS and HD for AC, BH and FB, respectively.

## 4 Discussion

Here we present the results of a feasibility study quantifying inter-observer variability in delineation of three critical abdominal OAR (liver, duodenum, stomach) across three motion management strategies: abdominal compression, breath-hold, and free breathing. Overall, there was no clear superiority of a single motion management strategy after geometric evaluation of delineated contours, nor of perceptual image quality. Inter-observer variation was below 2.3 mm for all OAR and motion management strategies, and intra-observer variation found to be largest in FB. This work revealed several challenges, underscoring the complex nature of the impact of motion management strategies on OAR delineation.

A key strength of this study includes the unique dataset of scans using multiple motion management strategies in each imaging session from the same participants providing intra-patient comparison. All minimum requirements for inter-observer studies as outlined by Guzene et al [25] were met in this study. This study is also strengthened by having four observers from a mixture of professional backgrounds, the fact that observers were blinded as much as possible to the motion management strategy when delineating OAR, and that multiple contour similarity metrics were evaluated. However, several challenges conducting a study of this type were identified. These warrant consideration, such as limited observer availability, which restricted the sample size of cases per motion management strategy to ensure the study's completion within a reasonable time. Incidence of liver and pancreas cancer is relatively rare [26], resulting in a smaller available sample size. This, in addition to the small variation effect size in geometric and perceptual image quality results across strategies mean that drawing conclusive findings from this study is challenging. Additionally, while scans were anonymised and the sequence randomised, there remains a small chance of observers identifying AC scans as the device’s influence on patient shape can sometimes be seen at the inferior end of the imaging field of view. Finally, due to the variation in participants, including a mixture of patient and non-patient participants and treatment sites, dosimetric evaluation of the impact of inter-observer variation was not evaluated.

There are few data published on OAR delineation variability, particularly in the abdomen, with much of the focus being on target delineation. Overall, inter-observer variability in our study was largest for the duodenum, whereas on motion-compensated and BH MRI in another study, agreement was lowest for liver [21], highlighting the potential impact of MRI sequence selection, as well as expertise of observers. The maximum HD presented in this work is a common metric for evaluation of contouring in radiotherapy but may be emphasizing worst-case discrepancies as HD is sensitive to outliers [13], [27]. Therefore, the 95 % HD may be more robust to outliers in future work, although this may not be simple or possible to compute in all TPS without external scripting. Additionally, much of the available published literature presents Dice similarity coefficient (DSC) values only, significantly limiting the possibility of comparison. DSC is a volume-based overlap metric which lacks spatial sensitivity [25] and is considered of limited use in practice. A study of interobserver variation in BH lung OAR delineation on CT showed that dosimetric impacts on OARs can be considerable, especially when considering steep SABR dose gradients [28]. This is relevant considering the proximity of sensitive OARs in liver or pancreatic SABR. It should also be noted that individual patient factors or tumour location may affect respiratory motion magnitude, and in turn MRI quality, however assessment of this was outside the scope of the current study.

In the present study, the duodenum consistently scored low on perceptual image quality across all motion management strategies and saw largest geometric variation. This may be in part due to the innate challenges of delineating this organ across imaging modalities [29] that the influence of other causes of physiological (i.e., peristaltic) motion-induced artefacts increases further away from the diaphragm. Additionally, intra-observer variation was larger than inter-observer for FB across all organs, particularly the duodenum, which may be due to factors including image quality and evolving interpretation of anatomy by observers over time and reflects the innate challenges of delineation of this organ.

BH, where the impact of respiratory motion is in theory removed, performed lower than anticipated in both perceptual image quality evaluation and geometric similarity to STAPLE contours. It should be noted that the comparison of in-phase reconstructions of T1W mDixon in BH with T2W sequences for AC and FB poses challenges due to their inherent differences in imaging techniques and tissue contrasts, which is both a limitation of the study but also of available technology. Additionally, delineation of the stomach on T2W MRI may make delineation of the stomach and duodenum easier as they tend to have a higher water content. Moreover, considering that the in-phase MRI generated from a T1W mDixon sequence in this study are not routinely used in our institution, observers may have been more familiar with reviewing T2W MRI, which may have influenced observers’ delineation proficiency. At our institution, planning and treatment verification of abdominal SABR using AC on the MR Linac are done using a motion-compensated 3D VANE radial stack-of-stars sequence. However, alternative sequences for delineation could be compared across AC and FB in the future, such as T2W 3D single-shot TSE. Acceleration techniques to reduce MRI acquisition times are a primary focus for vendors, and growing interest from the radiotherapy community could drive further development of specialized sequences. These include the motion-compensated periodically rotated overlapping parallel lines with enhanced reconstruction (PROPELLER) sequence discussed by Grimbergen et al, which was selected as the preferred sequence over 3D VANE and T2W MRI for delineation in online ART [30].

Interobserver delineation variation of OAR is one of many uncertainties within the treatment pathway to be accounted for, in addition to patient positioning variations, inter- and intra-fraction organ motion that can be mitigated with image-guided radiotherapy (IGRT) strategies [12], [31]. While evaluation of inter- and intra-fraction motion was outside of the scope of this study, it should be noted that clinically relevant inter-fraction organ deformation and baseline drifts of over 3 mm have been seen with AC [32] and intra-breath hold stability is a concern for abdominal radiotherapy delivery [33]. A planning organ at risk volume (PRV) margin of up to 5 mm is sometimes applied to the OAR in clinical practice, which is used to account for uncertainties in intra- and inter-fraction OAR position [34], although the size and use vary on an institutional basis based on local systematic and random errors per patient group. However, the future of PRV margins is unknown with the advent of real-time MR-guidance, therefore PRV use should not be used to account for uncertainties in delineation that can be mitigated with education and technology.

The sample size used in this study was based on available imaging data with all three motion management strategies and number of observers with experience in abdominal delineation. Future studies should include a case and observer sample size that is statistically powered to detect the desired level of reproducibility [25], however the results of the present study show minor differences, so a larger sample size is not expected to change results. Further evaluation of intra-observer variation would be advantageous in future studies, and it is advisable to utilize multiple rather than relying on a single repeat case. Future studies should incorporate observers from multiple institutions to externally validate results and increase generalizability, incorporate a training element to improve consistency, and use image sequences that have been further optimised to minimize potential factors affecting analysis.

As in other areas of radiotherapy, artificial intelligence (AI) solutions are seeing growing utilization for auto-segmentation to improve consistency, accuracy, and speed of delineation [35]. AI was not used for comparison in this study as a MR-based abdominal solution was not available, however, this should be included in future studies to evaluate current AI practice. While AI auto-segmentation for abdominal OAR is a promising development, many solutions require manual delineations and large datasets for algorithm training, which may be challenging in rarer abdominal cancers.

In conclusion, this study evaluated the inter-observer OAR delineation variation for three abdominal OAR on MR across three motion management strategies, highlighting challenges related to observers’ time, image quality, and dataset size. No single motion management strategy demonstrated superiority for all organs, emphasizing the need for tailored approaches based on tumour location. More work is needed to evaluate interobserver variation in abdominal OAR delineation, particularly regarding AI. Future studies should incorporate a larger number of observers, a training element, and optimized MR images.

## Funding

Mairead Daly is supported by Cancer Research UK RadNet Manchester [C1994/A28701], the Advanced Radiotherapy Technologies Network (ART-NET) [C309/A21993], the National Institute for Health and Care Research (NIHR) Manchester Biomedical Research Centre (BRC) (NIHR203308), and The Christie Hospital Charitable Fund. Ananya Choudhury, Alan McWilliam, Lisa McDaid and Cynthia Eccles are supported by NIHR Manchester Biomedical Research Centre (NIHR203308). The research was carried out at the National Institute for Health and Care Research (NIHR) Manchester Biomedical Research Centre (BRC) (NIHR203308). This work is also supported by Cancer Research UK Manchester Centre (CTRQQR-2021\100010). All authors declare no other conflicts of interest.

## 6 Clinical trial information

Quantification of Abdominal Organ Motion Using MRI (QUANTUM), NCT04748094. https://clinicaltrials.gov/study/NCT04748094.

## 7 Data sharing

Anonymized research data are stored in an institutional repository and can be shared upon formal request to the corresponding author.

## Declaration of competing interest

The authors declare the following financial interests/personal relationships which may be considered as potential competing interests: Mairead Daly reports financial support was provided by Cancer Research UK. All authors reports financial support was provided by Cancer Research UK. Mairead Daly reports financial support was provided by NIHR Manchester Biomedical Research Centre. Ananya Choudhury reports financial support was provided by NIHR Manchester Biomedical Research Centre. Alan McWilliam reports financial support was provided by NIHR Manchester Biomedical Research Centre. Lisa McDaid reports financial support was provided by NIHR Manchester Biomedical Research Centre. Cynthia Eccles reports financial support was provided by NIHR Manchester Biomedical Research Centre. Mairead Daly reports financial support was provided by The Christie Charity. If there are other authors, they declare that they have no known competing financial interests or personal relationships that could have appeared to influence the work reported in this paper.
